# Fast and accurate quantitative organic acid analysis with LC-QTOF/MS facilitates screening of patients for inborn errors of metabolism

**DOI:** 10.1007/s10545-017-0129-0

**Published:** 2018-02-12

**Authors:** Irene M. L. W. Körver-Keularts, Ping Wang, Huub W. A. H. Waterval, Leo A. J. Kluijtmans, Ron A. Wevers, Claus-Dieter Langhans, Camilla Scott, Daphna D. J. Habets, Jörgen Bierau

**Affiliations:** 1 Department of Clinical Genetics, Maastricht University Medical Centre, Maastricht, The Netherlands; 2 Department of Human Biology, NUTRIM School of Nutrition and Translational Research in Metabolism, Maastricht University Medical Centre, Maastricht, The Netherlands; 3 Translational Metabolic Laboratory, Department of Laboratory Medicine, Radboud University Medical Center, Nijmegen, The Netherlands; 4 Metabolic Laboratory, Center for Metabolic Diseases, University Children’s Hospital, Heidelberg, Germany; 5 Department of Newborn Screening, Clinical Chemistry, Sheffield’s Children’s Hospital, Sheffield, UK

## Abstract

Since organic acid analysis in urine with gaschromatography-mass spectrometry (GC-MS) is a time-consuming technique, we developed a new liquid chromatography-quadrupole time-of-flight mass spectrometry (LC-QTOF/MS) method to replace the classical analysis for diagnosis of inborn errors of metabolism (IEM). Sample preparation is simple and experimental time short. Targeted mass extraction and automatic calculation of z-scores generated profiles characteristic for the IEMs in our panel consisting of 71 biomarkers for defects in amino acids, neurotransmitters, fatty acids, purine, and pyrimidine metabolism as well as other disorders. In addition, four medication-related metabolites were included in the panel. The method was validated to meet Dutch NEN-EN-ISO 15189 standards. Cross validation of 24 organic acids from 28 urine samples of the ERNDIM scheme showed superiority of the UPLC-QTOF/MS method over the GC-MS method. We applied our method to 99 patient urine samples with 32 different IEMs, and 88 control samples. All IEMs were unambiguously established/diagnosed using this new QTOF method by evaluation of the panel of 71 biomarkers. In conclusion, we present a LC-QTOF/MS method for fast and accurate quantitative organic acid analysis which facilitates screening of patients for IEMs. Extension of the panel of metabolites is easy which makes this application a promising technique in metabolic diagnostics/laboratories.

**Electronic supplementary material:**

The online version of this article (10.1007/s10545-017-0129-0) contains supplementary material, which is available to authorized users.

## Introduction

Urinary organic acid analysis is a pivotal technique in selective screening for inborn errors of metabolism (IEMs) (Tanaka et al 1980; de Almeida and Duran 2014). The current state of the art relies on gas-chromatography coupled to mass spectrometry (GC-MS) of derivatized compounds. By its nature, the literally hundreds of organic acids present in human urine, endogenous as well as microbiome, drugs and other xenobiotic-derived metabolites (Blau et al 2014), can be detected by GC-MS. In fact, GC-MS analysis is the forerunner of untargeted metabolomics analysis as we nowadays envision it.

GC-MS has several advantages. It has high separation efficiency of metabolites, high specificity and sensitivity, few matrix effects, and broadly covered mass spectra libraries for identification of metabolites of interest are widely available (Pasikanti et al 2008). Disadvantages are clearly present since organic acids are not volatile and require organic solvent extraction and derivatization prior to GC-MS analysis. This makes the procedure laborious, and compared to underivatized tandem-mass spectrometry methods, time-consuming in terms of analytical run-time. Moreover, a relatively large sample volume is needed, despite efforts to increase throughput and reduce sample volume (Nakagawa et al 2010).

High resolution proton nuclear magnetic resonance (NMR) spectroscopy is a good alternative for organic acid analysis (and even broader IEM screening) with minimal sample preparation and a short experimental time. NMR spectroscopy did, however, not evolve in common IEM screening because of financial constraints and its relatively low sensitivity in the low millimolar range (Moolenaar et al 2003). Alternative liquid chromatography (LC)-quantitative hyphenated tandem mass spectrometry (MS/MS) techniques have been developed that allow high-throughput (Want et al 2010; Bouatra et al 2013). LC-MS/MS has outstanding sensitivity (in low nanomolar range) and specificity but only targeted metabolites, i.e., those a-priori selected in the method, are detected and quantified. The relatively recent introduction of high-resolution (HR) mass spectrometry in the form of time-of flight (TOF) MS and Orbitrap MS specificity allowed a major breakthrough. LC-HR MS combines the analytical power of LC-MS/MS with the unbiased quality of classical GC-MS, and thus enables not only the quantification of target metabolites, but also facilitates untargeted metabolite screening. Until now, LC-HR MS, including LC-QTOF/MS that combined with a quadrupole (Q), has been widely deployed in research settings (Paglia et al 2012), including inborn errors of metabolism (Wikoff et al 2007). Apart from a qualitative untargeted metabolomics approach (Miller et al 2015), no quantitative application suitable for routine diagnostic setting has been reported in the inborn errors of metabolism (IEM) field.

Here, we present our newly developed LC-QTOF/MS method for the quantitative analysis of urinary organic acids. This method covers a panel of critical biomarkers targeting defects in branched-chain amino acid-, lysine- and tryptophan-, aromatic amino acid-, neurotransmitter-, fatty acid- and pyrimidine metabolism as well as disorders in the Krebs cycle and urea cycle, amino acylase deficiencies and various other disorders. In addition, four medication-related metabolites were included in the panel, to allow discrimination between metabolic defects and medication. Analytical and diagnostic suitability were demonstrated in a cohort of individual urine samples, including proven IEMs. To expedite clinical interpretation we introduced z-score value plots. Our results demonstrate the suitability of this new method in the routine setting of selective metabolic screening.

## Material and methods

### Samples collection

All procedures followed were in accordance with the ethical standards of the “Human tissue and medical research- Code of Conduct for Responsible Use” by Dutch Federation of Biomedical Scientific Societies.

#### Clinical validation samples

Random urine specimens used in this study were selected from multiple laboratories. The specimens without a diagnosis (*N* = 88) used in this study were selected from the archive of the Maastricht Laboratory of Clinical Genetics. Samples were analyzed using the presently described method 1–2 years after routine diagnostic work-up and stored at −20 °C. Only urine samples from patients without a confirmed inborn error of metabolism (IEM) or any other biochemical finding or condition that was likely to influence the biochemical read-out were included. Samples of patients (*N* = 99) with confirmed diagnoses of IEM were included as positive controls. Detailed information on the clinical situation or treatment was not always available. These samples were from the archives of Maastricht Laboratory of Clinical Genetics, Translational Metabolic Laboratory, Radboud University Medical Centre, Nijmegen and from the archives of the Heidelberg and Sheffield qualitative organic acid assessment schemes of ERNDIM (European Research Network for evaluation and improvement of screening, diagnosis and treatment of Inherited disorders of Metabolism; www.erndim.org).

#### Method comparison samples

We used 28 urine samples of the ERNDIM quantitative organic acids quality assurance scheme from the period 2009–2015 for method cross validation.

### Chemicals, standards, and internal standards

All mobile phase solutions were prepared with UPLC-MS grade solvents of water, formic acid, and acetonitrile from Biosolve (Valkenswaard, the Netherlands). Supplemental Table S1 shows the 68 organic acid standards and 19 internal standards (ISTD) and their source. In the case of 2-pyrroloylglycine, hawkinsin, and 3-hydroxysebacic acid, urine samples of diagnosed patients with hyperprolinemia type II (HYRPRO2, OMIM #239510), hawkinsinuria (OMIM #140350), and long-chain 3-hydroxyl-CoA dehydrogenase deficiency (LCADD, OMIM #609016), respectively, served as surrogate standards. The four metabolites of medication were based on urine samples of patients using paracetamol (acetaminophen, glucuronide, and acetaminophensulphate), levetiracetam, and valproic acid (valproic acid glucuronide).

### LC-QTOF/MS method

Frozen urine samples were thawed at 37 °C and homogenized by vortex mixing; 25 μl urine adjusted to a creatinine concentration of 0–2 mM was mixed with 350 μL 0.1% *v*/v formic acid in water and 25 μL ISTD mixture. The samples were analyzed on an LC-QTOF/MS system (Agilent Technologies, Amstelveen, the Netherlands) that consisted of an Infinity II 1290 UHPLC coupled to a 6550 iFunnel QTOF equipped with an electrospray ionization source. The temperature in the multi-sampler was set at 4 °C. LC-separation was performed on an Acquity C18 column UPLC HSS T3 1.8 μm 2.1 × 100 mm with Acquity VanGuard PreColumn UPLC HSS T3; 2.1 × 5 mm (Waters, Manchester, UK) at 22 °C. The injection volume was 2 μL, followed by standard needle wash. The mobile phases for the reversed-phase (RP)-LC consist of solution A) 0.1% *v*/v formic acid in water and solution B) 0.1% formic acid in 95% acetonitril/5% water. The gradient program of the 35 min-cycle is described in Suppl. Table S2. The MS was tuned for low mass range (up to 1700 m/z) at high resolution slicer mode + 2G Hz extended dynamic range, and run in the negative mode for full scan with parameters listed in Suppl. Table S3. Agilent reference mass solution (containing reference compounds with m/z 112.9856 and m/z 1033.9881) was infused into the MS via a 1260 isocratic pump (Agilent) for continuous mass correction.

Calibration mixture stock solutions of 68 analytes were prepared and stored at −80 °C. In each batch, 6-point calibration curves were made with freshly prepared dilutions (Table 1). The quality of the batch was continuously monitored with a quality control (QC) sample with known amounts of 57 analytes. A QC sample and blank were injected at the start and after every 24th sample throughout the analytical workflow.

**Table 1:** Targeted biomarker panel

No.	Designation	Name	HMDB ID	[M-H]- ion, m/z	RT, min	ISTD	Calibration range, μM
**Disease markers**							
1		Acetoacetic acid	HMDB00060	101.0244	1.90	D3-propionylglycine	15.6–249.6
2	N-	Acetylglutamine	HMDB06029	187.0724	1.18	13C5-oxoglutaric acid	9.4–150.1
3	N-	Acetyl-L-alanine	HMDB00766	130.0510	2.62	13C4–3-hydroxybutyric acid	9.9–158.5
4	N-	Acetyl-L-aspartic acid	HMDB00812	174.0408	1.25	D3-propionylglycine	8.0–128.5
5	N-	Acetyl-L-methionine	HMDB11745	190.0543	5.19	D4-adipic acid	10.0–159.5
6		Adipic acid	HMDB00448	145.0506	4.93	D4-adipic acid	12.5–200.4
7		Argininosuccinic acid	HMDB00052	289.1154	0.72	D2-glycolic acid	6.9–111.0
8	N-	Butyrylglycine	HMDB00808	144.0666	4.22	D4-glutaric acid	0.5–7.9
9		Citric acid	HMDB00094	191.0197	1.88	D4-citric acid	331.0–5290.0
10		Ethylmalonic acid	HMDB00622	131.035	4.58	D5-ethylmalonic acid	8.7–138.6
11		Fumaric acid	HMDB00134	115.0037	2.06	D3-propionylglycine	8.3–133.3
12		Glutaric acid	HMDB00661	131.035	4.11	D4-glutaric acid	8.6–138.4
13		Glyceric acid	HMDB00139/HMDB06372	105.0193	0.76	D3-glyceric acid	8.0–128.2
14		Glycolic acid	HMDB00115	75.0088	0.78	D2-glycolic acid	19.9–318.4
15		Hawkinsin	HMDB02354	291.0777	1.16	D3-malic acid	▬
16		Hexanoylglycine	HMDB00701	172.0979	6.86	D3-hexanoylglycine	0.4–6.7
17		Homogentisic acid	HMDB00130	167.035	4.54	D4-sebacic acid	2.4–38.0
18		Homovanillic acid	HMDB00118	181.0506	6.14	D3-hexanoylglycine	6.2–99.7
19		Hydantoin-5-propionic acid	HMDB01212	171.0411	3.05	D3-methylmalonic acid	5.1–82.0
20	2-	Hydroxy-3-methylbutyric acid	HMDB00407	117.0557	4.76	D4-adipic acid	6.8–108.8
21	3-	Hydroxy-3-methylglutaric acid	HMDB00355	161.0455	3.68	D3–3-hydroxy-3-methyl-glutaric acid	10.8–172.8
22	2-	Hydroxy-3-methylpentanoic acid	HMDB00317	131.0714	6.03	D4-sebacic acid	6.1–97.0
23	2- & 3-	Hydroxyadipic acid	HMDB00321/HMDB00345	161.0455	3.73	D4-adipic acid	5.3–84.7
24	3-	Hydroxybutyric acid	HMDB00357	103.0401	2.56	13C4–3-hydroxybutyric acid	8.0–128.2
25	4-	Hydroxybutyric acid	HMDB00710	103.0401	1.93	13C4–3-hydroxybutyric acid	8.0–128.2
26	3-	Hydroxyglutaric acid	HMDB00428	147.0299	1.71	D3-propionylglycine	7.3–116.4
27	2-	Hydroxyglutaric acid	HMDB00606/HMDB00694	147.0299	1.40	D3-propionylglycine	7.3–116.4
28	5-	Hydroxyindoleacetic acid	HMDB00763	190.051	5.71	D3-hexanoylglycine	9.1–146.0
29	3-	Hydroxyisobutyric acid	HMDB00336/HMDB00023	103.0401	2.90	13C4–3-hydroxybutyric acid	12.2–195.4
30	2-	Hydroxyisocaproic acid	HMDB00746	131.0714	6.15	D4-adipic acid	8.8–141.3
31	3-	Hydroxyisovaleric acid	HMDB00754	117.0557	4.13	D4-glutaric acid	7.6–122.0
32	Ortho-	Hydroxyphenylacetic acid	HMDB00669	151.0401	6.64	D3-hexanoylglycine	8.1–129.6
33	4-	Hydroxyphenylacetic acid	HMDB00020	151.0401	5.81	D3-hexanoylglycine	8.1–129.6
34	4-	Hydroxyphenyllactic acid	HMDB00755	181.0506	5.17	D4-adipic acid	7.3–116.0
35	4-	Hydroxyphenylpyruvic acid	HMDB00707	179.035	4.62	D4-adipic acid	8.4–135.1
36	3-	Hydroxypropionic acid	HMBD00700	59.0133*	1.18	13C3-lactic acid	6.9–111.0
37	3-	Hydroxysebacic acid	HMDB00350	217.1081	6.69	D4-sebacic acid	▬
38		Isobutyrylglycine	HMDB00730	144.0666	4.12	D4-adipic acid	0.4–5.7
39		Isovalerylglycine	HMDB00678	158.0823	5.16	D3-hexanoylglycine	0.7–11.3
40	alpha-	Ketoisovaleric acid	HMDB00019	115.0401	3.81	D4-adipic acid	8.0–128.1
41		Lactic acid	HMDB00190	89.0244	1.18	13C3-lactic acid	50.3–804.2
42		Malic acid	HMDB00744	133.0142	1.04	D3-malic acid	7.9–125.7
43		Malonic acid	HMDB00691	103.0037	1.18	D3-propionylglycine	7.1–113.7
44	3-	Methyl-2-oxovaleric acid	HMDB00491	129.0557	5.25	D4-adipic acid	8.5–135.3
45	2-	Methyl-3-hydroxybutyric acid	HMDB00354	117.0557	4.40	D4-adipic acid	7.1–114.0
46	3-	Methyladipic acid	HMDB00555	159.0663	5.76	D4-adipic acid	7.5–120.6
47	2-	Methylbutyrylglycine	HMDB00339	158.0823	5.03	D3-hexanoylglycine	0.7–11.0
48	2-	Methylcitric acid	HMDB00379	205.0354	3.92	D3-methyl citric acid	7.9–126.0
49	3-	Methylcrotonylglycine	HMDB00459	156.0666	5.17	D3-hexanoylglycine	0.6–9.0
50	3-	Methylglutaconic acid	HMDB00522	99.0452 *	4.84	D4-adipic acid	7.9–125.8
51	3-	Methylglutaric acid	HMDB00752	145.0506	5.00	D4-adipic acid	8.8–141.6
52		Methylmalonic acid	HMDB00202	117.0193	2.89	D3-methylmalonic acid	8.1–128.9
53		Methylsuccinic acid	HMDB01844	131.035	4.49	D5-ethylmalonic acid	5.1–81.0
54		Mevalonic acid	HMDB00227	147.0663	3.15	D3-methylmalonic acid	13.0–210.0
55		Orotic acid	HMDB00226	155.0098	1.18	D2-glycolic acid	6.7–107.1
56		Oxoadipic acid	HMDB00225	159.0299	2.34	D4-sebacic acid	8.1–129.9
57		Oxoglutaric acid	HMDB00208	145.0142	1.19	13C5-oxoglutaric acid	20.2–323.0
58	3-	Phenyllactic acid	HMDB00748	165.0557	6.95	D3-hexanoylglycine	7.2–114.5
59		Phenylpropionylglycine	HMDB00860	206.0823	7.40	D3-hexanoylglycine	0.4–6.1
60		Propionylglycine	HMDB00783	130.051	2.33	D3-propionylglycine	0.5–8.6
61		Pyroglutamic acid	HMDB00267	128.0353	1.84	D5-pyroglutamic acid	6.2–99.8
62	2-	Pyrroloylglycine	HMDB59778	167.0462	4.61	D5-ethylmalonic acid	▬
63		Pyruvic acid	HMDB00243	87.0088	0.91	13C3-pyruvic acid	6.1–97.2
64		Sebacic acid	HMDB00792	201.1132	9.05	D4-sebacic acid	1.6–25.6
65		Suberic acid	HMDB00893	173.0819	6.85	D4-sebacic acid	5.5–88.0
66		Suberylglycine	HMDB00953	230.1034	5.68	D3-hexanoylglycine	0.4–6.3
67		Succinic acid	HMDB00254	117.0193	2.55	D3-methylmalonic acid	31.0–495.6
68		Succinylacetone	HMDB00635	157.0506	4.50	13C5-Succinyl aceton	6.7–107.5
69		Tiglylglycine	HMDB00959	156.0666	5.07	D3-hexanoylglycine	0.9–14.3
70		Vanillactic acid	HMDB00913	211.0612	5.51	D4-adipic acid	7.7–123.4
71		Vanillylmandelic acid	HMDB00291	197.0455	4.05	D4-glutaric acid	8.6–138.1
**Medication markers**							
72		Acetaminophen glucuronide	HMDB10316	326.0881	4.19	D4-glutaric acid	▬
73		Acetaminophensulphate	HMDB59911	230.0129	4.62	D5-ethylmalonic acid	▬
74		Levetiracetam	HMDB15333	202.0721	4.54	D5-ethylmalonic acid	▬
75		Valproic acid glucuronide	HMDB00901	319.1398	9.05	D4-sebacic acid	▬

Note: * in [M-H]- ion indicates used fragment ion that comes from in source fragmentation

### Data analysis and statistics

MassHunter workstation software (Agilent) Acquisition B.06 and Quantitative Analysis for Q-TOF B.07 were used for MS data acquisition and analysis. The monitored ions and their retention times, established with standards or patients samples, and the ISTD of each analyte are listed in Table 1. The peak area was used for quantification. Each analyte was absolutely quantified based on its own standard calibration curve, except the seven aforementioned analytes based on patients’ urine, of which concentrations were calculated with a relative ISTD method assuming the analyte has the same response factor as its ISTD. All concentrations were normalized to urine creatinine concentrations obtained from clinical chemistry lab (Jaffe method).

Z-scores were calculated by comparing analyte log transformed values to the associated mean and standard deviation found in a control cohort without a diagnosed IEM (*n* = 46) in two age groups (0–2 years and >2 years). Missing values and values below the estimated limit of detection (LOD, concentration equivalent to 3 * S/N) were imputed with LOD before log transformation. For the analytes that were not detected in normal urines, SD was set as 0.774 to report a z-score value of 3 when the measurement result is five times the LOD.

### Method analytical validation

The method was validated according to our internal validation procedure for quantitative methods based on the Dutch NEN-EN-ISO 15189 guide “Medical Laboratories-Requirements for quality and competence” (Nederlands Normalisatie-instituut (NEN) 2012).

### Method cross validation

Urine samples from the ERNDIM Quantitative Organic Acids External Quality Assessment Scheme were analyzed by GC-MS and the UPLC-QTOF/MS method. The results from GC-MS analyses as performed in the ERNDIM scheme were used as a reference for the new method. The results of the medians of participants in the ERNDIM scheme were retrieved from ERNDIM archive; 98% of the participants used GC-MS (Peters et al 2016).

We compared the results of 24 spiked analytes in 28 ERNDIM samples on recovery and linearity (Martens and Weykamp 2016). Method differences were assessed per analyte by t-test or Mann-Whitney test when proper. A *P*-value <0.05 was considered to be significant. We also applied Passing-Bablok regression analysis (Passing and Bablok 1983). The above analyses were done in R environment, and with method comparison regression (MCR) package (Manuilova et al 2014; R Core Team 2016).

## Results

### Analytical characteristics of the LC-QTOF/MS method

The UHPLC coupled QTOF/MS method was able to separate and identify 74 analytes. The 2- and 3-hydroxyadipic acid isomers could not be resolved due to identical retention times and were therefore analyzed as one combined signal. Adipic acid and 3-methylglutaric acid have the same mass and comparable hydrophobicity. At normal physiological conditions, concentrations of both metabolites are low and there is a 0.05 min difference in retention time allowing adequate distinction. At pathological levels, however, the peak of the biomarker compound overwhelms another one. We need to combine the profile of biomarks and clinical presentation to resolve the identity of the peak. The suboptimal peak shapes of 2-methylcitric acid, succinylacetone (Suppl. Figure), citric acid, and oxoadipic acid hampered correct quantification, but analogue isotopic internal standards corrected for that to some extent.

#### Linearity and recovery

Linear calibration curves (*R*^2^ > 0.98) were obtained in water and urine for all 68 analytes (Table 1). The recovery was 100 ± 15% for the vast majority of analytes (63/68) in urine. For 2-methylcitric acid, acetoacetic acid, 4-hydroxybutyric acid, malonic acid, and pyroglutamic acid recovery was out of this range. For 2-methylcitric acid, acetoacetic acid, and malonic acid this can be explained by the chromatographic peak shape. Detection of 4-hydroxybutyric acid and pyroglutamic acid was complicated by ion suppression which could not be completely corrected for by using alternative internal standards.

#### Limits of detection and quantification

The majority of the analytes had an estimated limit of quantification (LOQ, concentration equivalent to S/*N* > 10) < 3 μmol/l. Succinic acid, oxoglutaric acid, ethylmalonic acid, 2-oxovaleric acid, 2-methyl-3-hydroxybutyric acid, 4-hydroxyphenylpyruvic acid, fumaric acid, glycolic acid, 3-methylglutaconic acid, malic acid, oxoadipic acid, 3-hydroxyisobutyric acid, 3-hydroxybutyric acid, succinylacetone, pyruvic acid, and methylmalonic acid had a LOQ ≤ 15 μmol/l. Three compounds had a higher LOQ, i.e., 4-hydroxybutyric acid (18 μmol/l), malonic acid (21 μmol/l), and acetoacetic acid (69 μmol/l).

#### Within- and between-run variation

Within-run variation was satisfactory for all analytes in urine, the coefficient of variation was ≤10% for the vast majority of compounds. The coefficient of variation was slightly higher for lactic acid (11%), 2-methylcitric acid (12%), malic acid (12%), and mevalonic acid (23%). For 2-methylcitric acid and malic acid the higher variation is explained by poor peak-shape, whereas mevalonic acid is unstable in the acid environment.

The between-run variation was satisfactory at low (CV < 20%) and for high calibration levels (CV < 10%) for the majority of compounds. For acetoacetic acid the CV was 13% at 93 μM; for 2-methylcitric acid CV 14% at 88 μM, poor peak-shape being the main explanation.

**Fig. 1:**
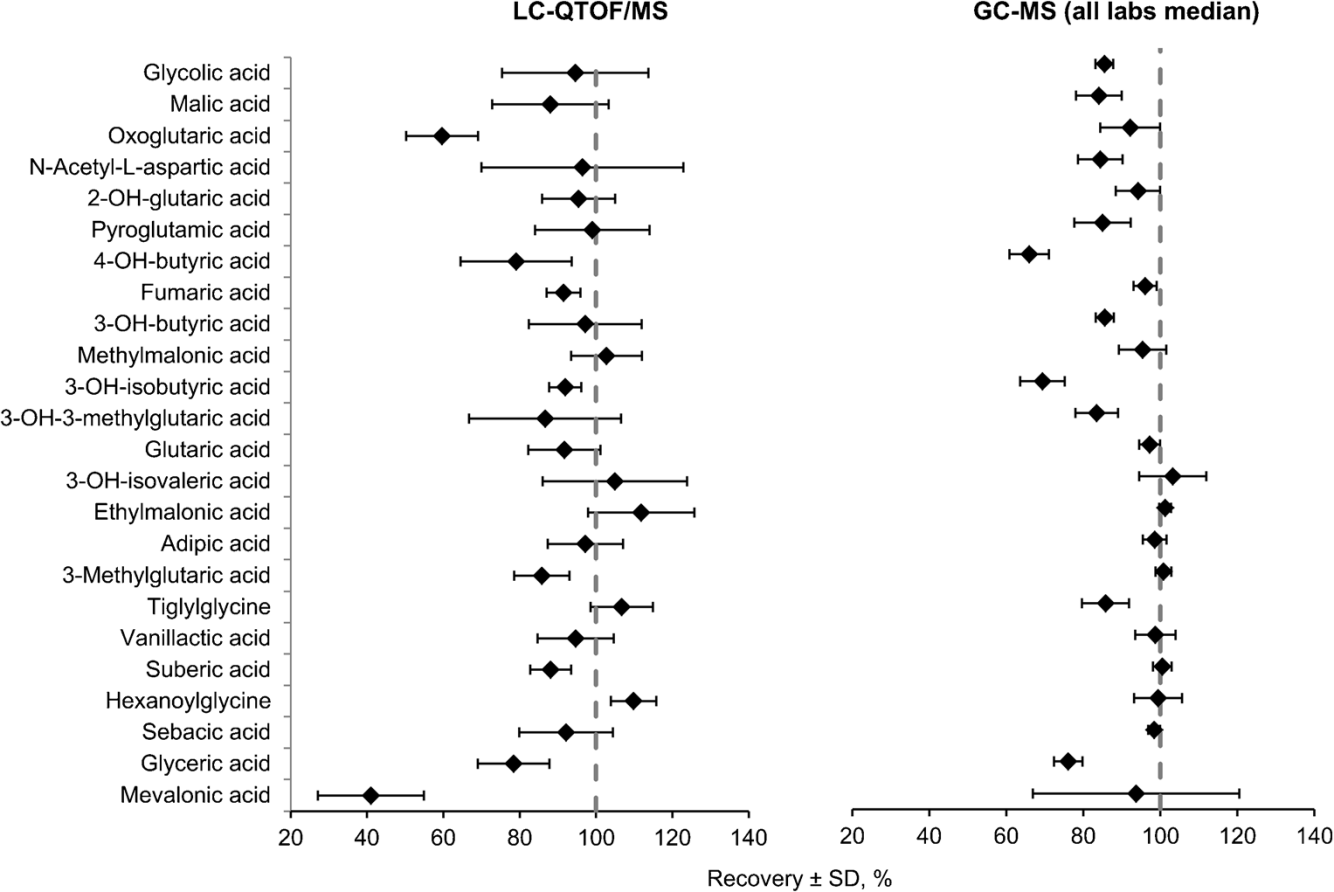
Comparison of the general GC-MS method with our new LC-QTOF/MS on 28 ERNDIM QC urine samples containing 24 spiked analytes

**Fig. 2:**
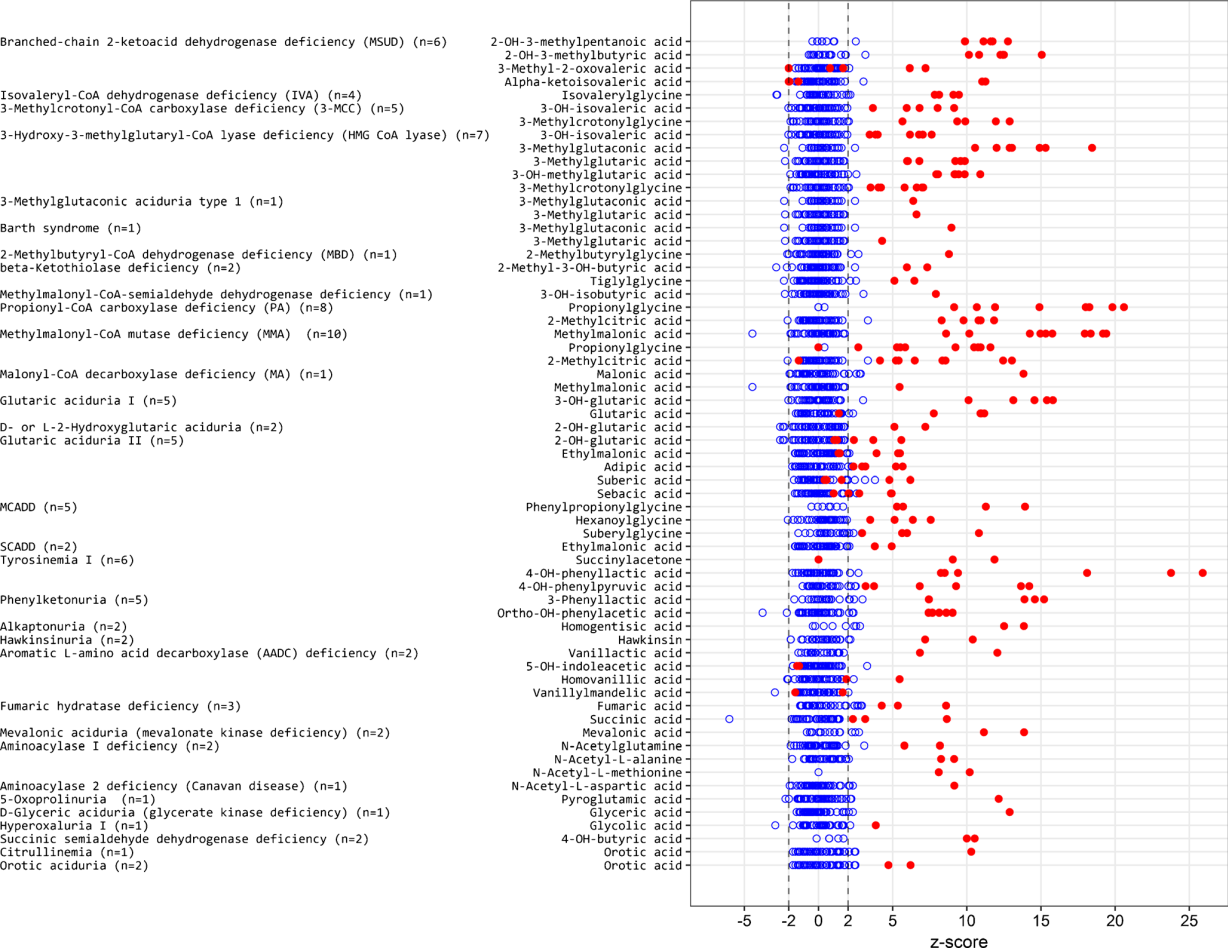
Summary of z-scores of critical biomarkers for 32 IEMs. Both urines from patients with an IEM as well as controls negatively tested for IEM (*n* = 46) were included. Red dot indicates a patient, blue circle is from a control

**Fig. 3:**
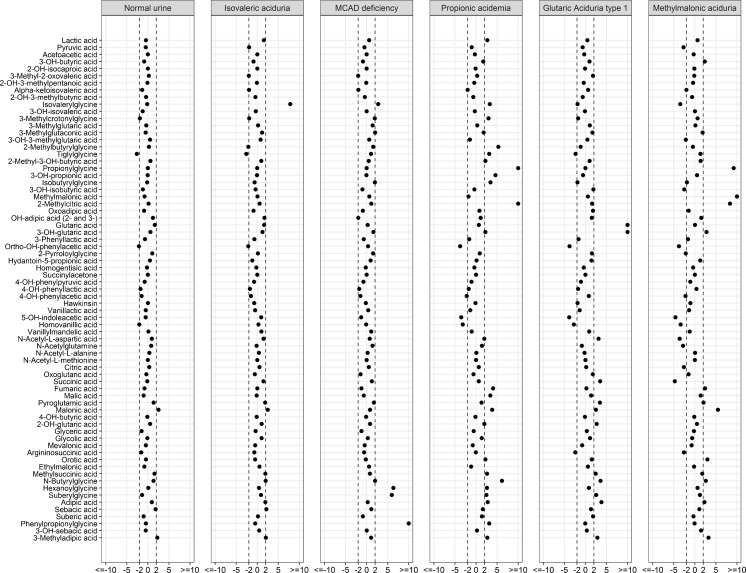
**Z-score profiles of some representative IEMs:** isovaleric aciduria (IVA, OMIM #243500), medium chain acyl-CoA dehydrogenase deficiency (MCADD, OMIM #201450), propionic aciduria (PA, OMIM #606054), glutaric aciduria type 1 (GA I, OMIM #231670), and methylmalonic aciduria (MMA, OMIM #251000)

1. **Comparison and correlation between GC-MS and LC-QTOF/MS methods**
  The results on 24 organic acids in 28 samples from the ERNDIM quantitative organic acids scheme were used to compare the GC-MS and LC-QTOF/MS methods (Fig. 1). An acceptable recovery for an individual metabolite was defined as a recovery between 80 and 120%. For the LC-QTOF/MS method 20/24 analytes showed an acceptable recovery which was similar to medians of all participating labs in the original ERNDIM scheme (21/24) (Fig. 1). LC-QTOF/MS had significantly improved recovery for: pyroglutamic acid, tiglylglycine, and 3-hydroxyisobutyric acid. For oxoglutaric acid and mevalonic acid, the LC-QTOF/MS recovery was below 80% but still satisfactory for diagnostic purposes. Both GC-MS (medians of all labs) and LC-QTOF/MS methods had excellent linearities for all 24 analytes (Suppl. Table s4). Passing-Bablok regression analysis indicated that the LC-QTOF/MS and the GC-MS (median of all labs) methods were highly correlated and comparable. Mevalonic acid, oxoglutaric acid, 3-hydroxyisobutyric acid, pyroglutamic acid, and tiglylglycine showed a significant difference with a slope out of range 0.8–1.2 (Suppl. Table S4).
2. **Clinical validation of the LC-QTOF/MS methods**
  In the clinical validation, we analyzed 99 diagnostic samples of patients with an IEM (32 different IEMs included) and 88 control samples. Figure 2 is a summary of the z-scores on critical biomarkers for the 32 different IEM. Most IEM were easily recognized with z-scores of biomarkers clearly >2.5 in the z-score profile. In our daily practice, the actual concentrations are considered in addition to the z-scores profile. In all control urine samples, the majority of the biomarkers had z-scores between −2.5 to 2.5. Figure 3 shows representative z-score profiles of patients with isovaleric aciduria (IVA, OMIM #243500), medium chain acyl-CoA dehydrogenase deficiency (MCADD, OMIM #201450), propionic aciduria (PA, OMIM #606054), glutaric aciduria type 1 (GA I, OMIM #231670), and methylmalonic aciduria (MMA (*mut*^*0*^), OMIM #251000).

For maple syrup urine disease (MSUD, OMIM #248600), 2-hydroxy-3-methylpentanoic acid and 2-hydroxy-3-methylbutyric acid were the most discriminating biomarkers with z-scores >10 in all patients. Five patients with a glutaric aciduria type I (GA I, OMIM #231670) were easily diagnosed based on elevated concentrations of 3-hydroxy-glutaric acid (z-score > 10); four of them also had significantly elevated glutaric acid. Only one GA I patient had a normal excretion of glutaric acid.

Five patients with glutaric aciduria type II (GA II, multiple acyl-CoA dehydrogenase deficiency (MADD), OMIM #231680) presented with an increased excretion of ethylmalonic acid (EMA), glycine conjugated as well as dicarboxylic acids. Glutaric acid and 2-hydroxyglutaric acid were not consistently elevated in all three cases. A mild MADD case even presented with non-significant excretion of 2-hydroxyglutaric acid (z-score 2.5) but with a clear increase in both EMA and dicarboxylic acids. The excretion of EMA in MADD is comparable in z-score to short-chain acyl-CoA dehydrogenase deficiency (SCADD, OMIM #201470).

In all MCADD patients, phenylpropionylglycine, hexanoylglycine, and suberylglycine were significantly increased with z-scores >5.0.

One patient with 5-oxoprolinuria (pyroglutamic aciduria, OMIM #266130) was included and had a high excretion of pyroglutamic acid (z-score > 10). The absence of paracetamol metabolites acetaminophen glucuronide and acetaminophensulphate excluded medication-related changes. In other patients within our sample panel on paracetamol indicated by the presence of these two medication-related metabolites, only a minor increase of pyroglutamic acid (z-score 2.5–5.0) (data not shown) was observed.

## Discussion

Urine organic acid analysis is a pivotal part in the diagnostic workup of inborn errors of metabolism (IEM). We developed a LC-QTOF/MS method for the quantification of 71 metabolites in urine covering all disorders classically identified through GC-MS analysis of organic acids. This targeted analyses is aimed at finding most organic acidurias in a fast manner, with the limitation that some IEM classically detected in GC-MS analysis, such as glycerol kinase deficiency, are not detected. Obviously, in case of strong clinical suspicion additional analyses are required in these cases. However, our method can be easily expanded by adding more relevant biomarkers for other IEM. The method was validated according to Dutch NEN-EN-ISO 15189 standards underlining its fit for purpose in the routine setting of a specialized diagnostic laboratory in the field of IEM. Performance characteristics (i.e., recovery) of our new analytical UHPLC-QTOF/MS assay were comparable or even superior to the classical GC-MS analysis. Cross validation showed on linearities and recoveries that our results compared well to that of the median of all labs for the GC-MS method. The targeted approach with internal standards makes our method robust. In addition, simple sample preparation and short time from unprocessed urine sample-to-authorized lab result of an individual sample (< 3–4 h) are clear benefits for the LC-QTOF/MS assay. However, for two metabolites, i.e., oxoglutaric acid and mevalonic acid, LC-QTOF/MS had a worse recovery than the classical assay. Two cases of classical mevalonic aciduria (MEVA, OMIM #610377) were easily diagnosed based on z-score profiles with abnormal mevalonic acid. Hyper-IgD syndrome (OMIM **#**260920) diagnostics depends on detecting more subtle increased excretion of mevalonic acid especially in periods of episodic fever (Prietsch et al 2003). We measure only mevalonic acid which is in equilibrium with lactone form (not detected in our method) in mild acid conditions; additional HIDS patient sample (especially in crisis) analysis would be required to evaluate the feasibility of these diagnostics with our new method.

We clinically validated our new assay by evaluating 32 different IEM and in total 187 samples with a representative number of control samples showed that this new diagnostic approach with z-score profiles facilitates diagnostics of the majority of organic acidurias. Most biomarkers associated with the IEMs included as reported in literature (Blau et al 2014) were observed in significantly elevated levels (z-score > 3.0) in the z-score plots for most IEM. Not all metabolites were increased to the same extent in all patients reflecting severity of the disease and clinical condition and/or treatment status (detailed information however was not always available). In patients with MSUD, not all markers were elevated in all patients; however, due to the presence of multiple biomarkers for MSUD in the metabolite panel, this diagnosis could not be missed. Glutaric acid was not elevated in all urine samples from GA I and MADD patients. With urine organic acid analysis, 3-hydroxyglutaric acid is the diagnostic metabolite. Glutaric acid can be completely normal in some patients but 3-hydroxyglutaric acid is pathognomonic for GA I. 3-hydroxyglutaric acid can be hard to identify and quantitate since it may co-elute with 2-hydroxyglutaric acid in GC-MS analyses (Hedlund et al 2006), depending on the analytical column. In our newly established LC-QTOF/MS assay, we could separate 2- and 3-hydroxyglutaric acid quite well, which allows unambiguous annotation and quantification of both metabolites.

All MCADD patients had abnormal z-score profiles. MCADD diagnostics is usually based on acylcarnitine profiling and organic acid analysis. Browning et al (2005) showed that normal acylcarnitine levels during confirmation of abnormal newborn screening have been encountered in some fatty acid oxidation disorders. Our findings are therefore of extra information on diagnostics. For these notoriously difficult IEMs several analyses (both acylcarnitine and organic acid analysis) in different settings (fasting and fed state) could be of added value in the diagnostics. Eventually, enzyme or gene analysis confirms a diagnosis based on biochemical abnormalities.

One patient with 5-oxoprolinase deficiency (pyroglutamic aciduria) could be easily discriminated on the basis of the z-score for secondary causes of 5-oxoprolinuria like a certain drug (paracetamol) (Saudubray et al 2016). The absence of paracetamol related markers in this patient supported the diagnosis. Other causes of 5-oxoprolinuria which include severe burns, inborn errors of metabolism not involving the gamma-glutamyl cycle, e.g., X-linked ornithine transcarbamylase deficiency, urea cycle defects, tyrosinemia, and homocystinuria were, however, not included in this evaluation.

All these factors and representation of results as a long list of numbers made it easy to miss a diagnosis despite reference values. This is why we introduced z-score plots. In the broad panel of IEMs included in this clinical validation, the z-score plot proved to be a useful and easy to use tool for labs to interpret the analytical results of the LC-QTOF/MS method.

The targeted biomarkers approach and number of IEM diseases that can be diagnosed can be continuously expanded to meet the complexity of IEM diagnostics. The full scan method is flexible to include new analytes and update the method to meet this challenge. This is part of our future work. We will explore not only the negative but also the positive ionization mode to cover broad chemical groups, including amino acids, acylcarnitines, purine and pyrimidine metabolites. Eventually, the method can be a generic metabolic screening method on the LC-QTOF/MS platform whereby urine is analyzed as a front-line specimen for screening of IEMs. This would provide a valuable means to efficiently, accurately, and rapidly identify and manage many IEMs (Campeau et al 2008).

## Electronic supplementary material

ESM 1 (XLSX 13.7 kb)

ESM 2 (XLSX 8.19 kb)

ESM 3 (XLSX 8.69 kb)

ESM 4 (XLSX 16.4 kb)

ESM 5 (PDF 602 kb)
