# Supplementary material for: Fast and accurate quantitative organic acid analysis with LC-QTOF/MS facilitates screening of patients for inborn errors of metabolism
Source: J Inherit Metab Dis. 2018 Feb 12;41(3):415–24. doi: 10.1007/s10545-017-0129-0 (PMC5959959; doi:10.1007/s10545-017-0129-0)
Supplement: Supplementary file 5 — (PDF 602 kb) [file 10545_2017_129_MOESM5_ESM.pdf]

Supplemental Figure: Typical chromatograms of peaks with poor peak shape or from isomers.

A. The separation of isomers 2-Hydroxyglutaric acid and 3-Hydroxyglutaric acid

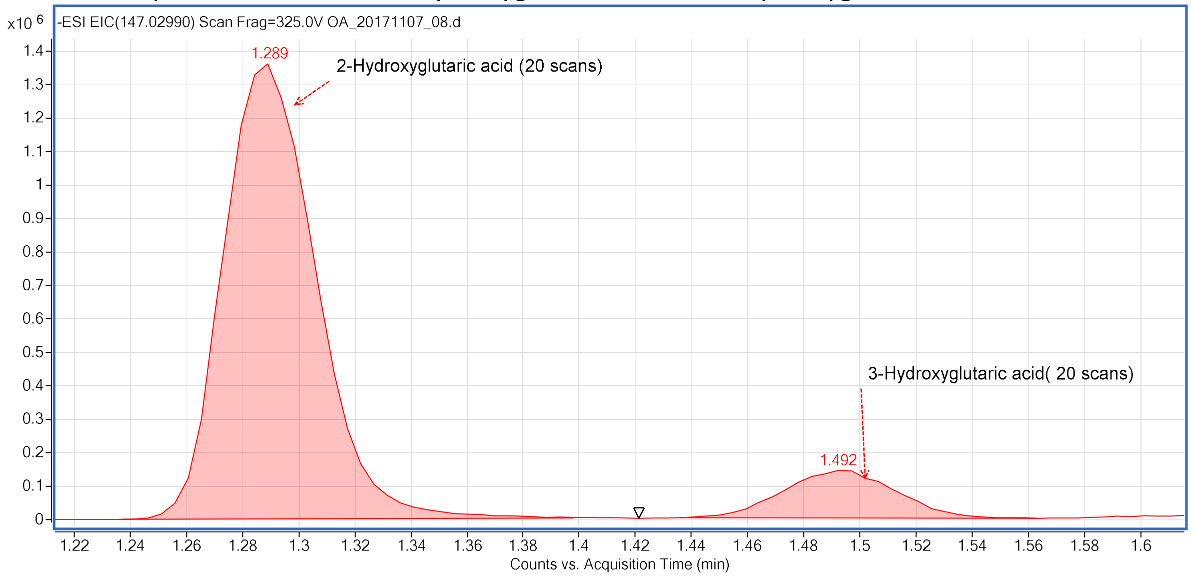

Standard 2-Hydroxyglutaric acid concentration is 153 µM; Standard 3-Hydroxyglutaric acid 58 µM

B: The multiple-peaks of 2-methylcitric acid

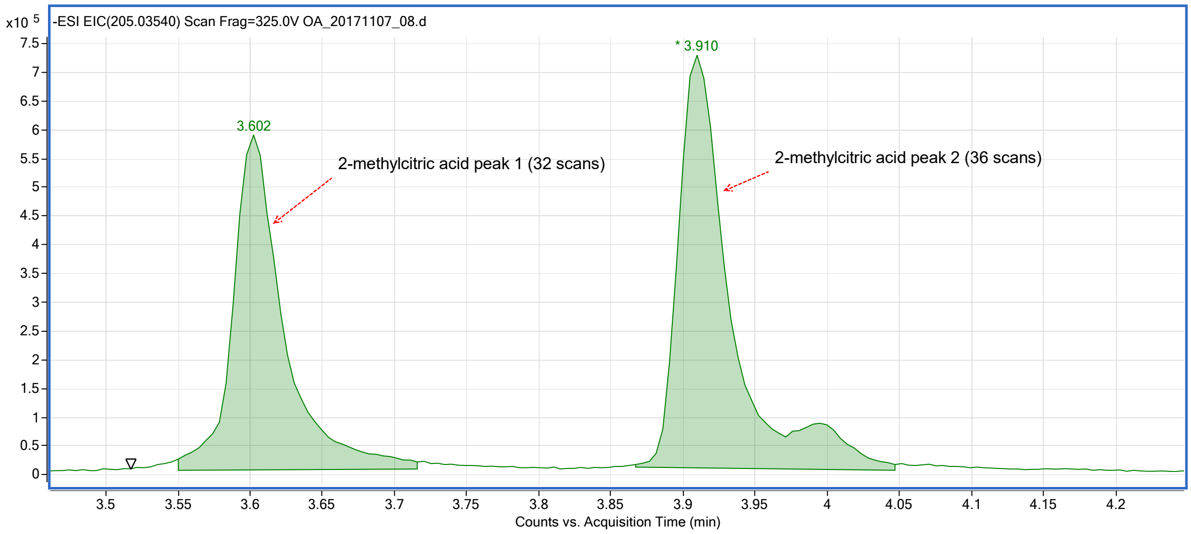

Standard 2-methylcitric acid concentration 150 µM

C: The poor peak shape of succinylacetone

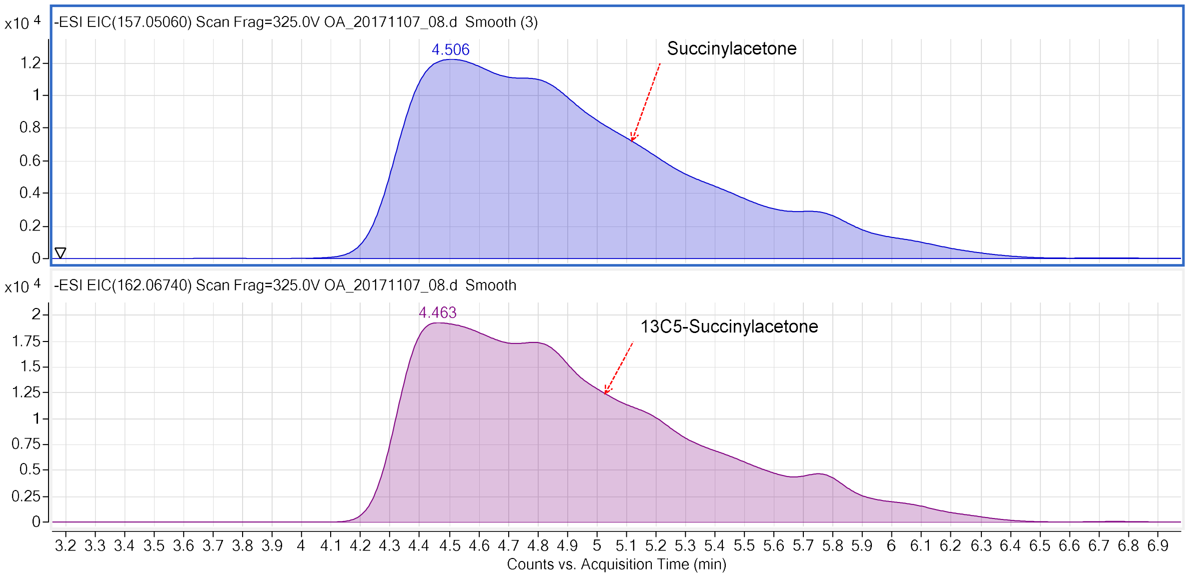

Standard Succinylacetone concentration 53 µM; Internal standard 13C5-Succinylacetone 75 µM
